# OBHS Drives Abnormal Glycometabolis Reprogramming via GLUT1 in Breast Cancer

**DOI:** 10.3390/ijms24087136

**Published:** 2023-04-12

**Authors:** Kexin Wang, Qiuzi Li, Yufeng Fan, Pingping Fang, Haibing Zhou, Jian Huang

**Affiliations:** 1 Hubei Key Laboratory of Cell Homeostasis, College of Life Sciences, Wuhan University, Bayi Road, Wuhan 430072, China; 2 Department of Pharmacology, School of Basic Medical Sciences, Wuhan University, Wuhan 430071, China; 3 State Key Laboratory of Virology, Frontier Science Center for Immunology and Metabolism, Hubei Province Engineering and Technology Research Center for Fluorinated Pharmaceuticals, School of Pharmaceutical Sciences, Wuhan University, Donghu Road, Wuhan 430071, China

**Keywords:** breast cancer, combination therapy, GLUT1, glycometabolism, OBHS

## Abstract

Due to the poor metabolic conditions fomenting the emergence of the Warburg effect (WE) phenotype, abnormal glycometabolism has become a unique and fundamental research topic in the field of tumor biology. Moreover, hyperglycemia and hyperinsulinism are associated with poor outcomes in patients with breast cancer. However, there are a few studies on anticancer drugs targeting glycometabolism in breast cancer. We hypothesized that Oxabicycloheptene sulfonate (OBHS), a class of compounds that function as selective estrogen receptor modulators, may hold potential in a therapy for breast cancer glycometabolism. Here, we evaluated concentrations of glucose, glucose transporters, lactate, 40 metabolic intermediates, and glycolytic enzymes using an enzyme-linked immunosorbent assay, Western blotting, and targeted metabolomic analysis in, in vitro and in vivo breast cancer models. OBHS significantly inhibited the expression of glucose transporter 1 (GLUT1) via PI3K/Akt signaling pathway to suppress breast cancer progression and proliferation. Following an investigation of the modulatory effect of OBHS on breast cancer cells, we found that OBHS suppressed the glucose phosphorylation and oxidative phosphorylation of glycolytic enzymes, leading to the decreased biological synthesis of ATP. This study was novel in highlighting the role of OBHS in the remodeling of tumor glycometabolism in breast cancer, and this is worth further investigation of breast cancer in clinical trials.

## 1. Introduction

Among women, breast cancer accounted for 31% of all new diagnoses in 2023 thus far. About 55,720 cases of female breast ductal carcinoma in situ will be diagnosed in 2023 [1]. Female breast cancer incidence rates have been increasing by about 0.5% per year since 2000s, and this trend has been attributed, at least in part, to continued increases in excess body weight [2,3]. Irregular sleep and poor diets are the main causes of hyperglycemia, and disordered glucose metabolism is an important risk factor for the subsequent development of many malignant tumors [4]. Epidemiologic evidence of a close relationship between type 2 diabetes and increased cancer risk and the mechanisms by which obesity and the dysregulation of the host’s metabolism may promote breast cancer have been reported many times [5,6,7,8,9]. The uncontrolled growth of tumors involves abnormal cell proliferation, as well as corresponding adjustments in energy metabolism to fuel cell growth and division [10,11]. Tumors consume more glucose than normal tissues do by preferentially converting it into lactate instead of oxidizing it in the mitochondria. The metabolism of glucose, the most central macronutrient, involves converting energy to be transformed to ATP. This activity is essential for sustaining the growth of all cells. In 1924, Warburg observed an increase in glucose uptake and lactate production in tumor cells compared with those in normal cells, even in the presence of an adequate amount of oxygen [12,13]; hence, the term “aerobic glycolysis” was coined. This metabolism of glucose into lactate, despite the presence of an adequate amount of oxygen, is called the Warburg effect (WE), which unknowingly laid the foundation for the field of cancer metabolism. Previous studies showed that the WE is a general metabolic switch accompanying tumor cell proliferation [14]. In breast cancer, combination therapy, including chemotherapy, tends to induce hyperglycemia, leading to poor outcomes in patients. Currently, the field of cancer glycometabolism in molecular biology and genetics has opened the door to new cancer therapies.

Oxabicycloheptene sulfonate (OBHS) functions as a novel selective estrogen receptor modulator (SERM) with estrogen receptor (ER) antagonistic activity, DNA damage activity, and anti-inflammatory activity [15,16,17,18]. However, not a lot is known about the glucometabolic remodeling action of OBHS in cancer. Therefore, we establish a comparison system using antidiabetic medication as the specific positive control group with the aim to more clearly figure out the molecular mechanisms of OBHS. Many preclinical studies have shown the anticancer effects of antidiabetic medication in breast cancer, and also in other types of cancer, especially 2-deoxy-D-glucose (2-DG) and metformin [19,20,21,22]. To form 2-deoxy-d-glucose-6-phosphate, 2-DG mimics D-glucose, which accumulates inside the cell and leads to the inhibition of glycolysis [23,24,25,26,27,28]. Metformin is widely used to treat type 2 diabetes by inhibiting mitochondrial complex I, and it has recently emerged as a potential anticancer agent [29,30,31,32]. Based on the mechanisms of action for these two drugs, we classify glycolysis into upper (glucose phosphorylation) and lower (oxidative phosphorylation) glycolysis, which helps us to study the role of OBHS in glycolysis and the related remodeling mechanisms. This information would be useful for providing potential therapeutic directions and identifying subsets of patients who are most likely to benefit from glycolytic targeted therapies.

## 2. Results

### 2.1. GLUT1 Expression Significantly Correlated with Poorer Survival Statistics among Patients with Breast Cancer in Clinical Settings

**Figure 1:**
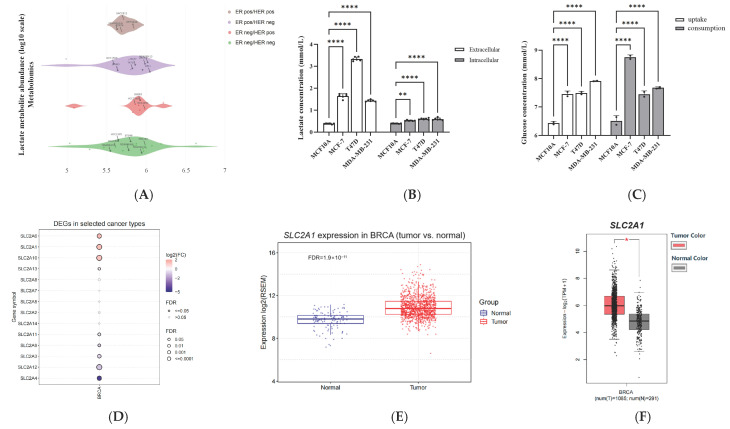

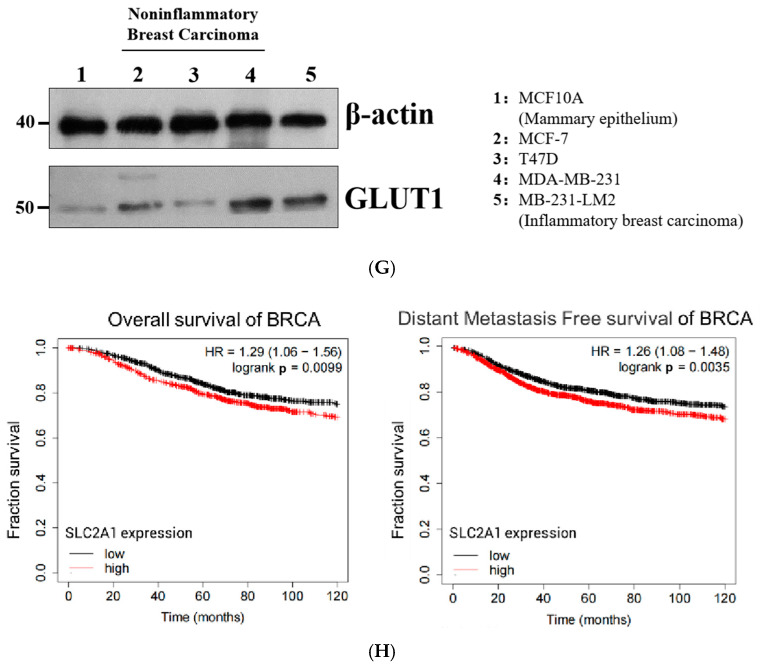
mRNA expression level of *SLC2As* in cancer tissues and subtypes of breast cancer correlated with low survival rate of patients. (**A**) High levels of lactate production in different subtypes of breast cancer calculated using the depmap portal. (**B**) Lactate concentration and (**C**) glucose uptake and glucose consumption in MCF10A, MCF-7, T47D, and MDA-MB-231 cells. (**D**) Differentially expressed genes (DEGs) between tumor and normal in BRCA. mRNA expression levels of *SLC2A1* in normal and breast cancer tissues calculated using (**E**) GSCA and (**F**) GEPIA database; * *p* < 0.05 versus normal tissue. (**G**) Protein lysates were subjected to Western blot analysis for the expressions of GLUT1 and β-actin in different subtypes of breast cancer. (**H**) Relationship between *SLC2A1* expression and OS and DMFS in breast cancer patients. (Plots showing low expression in black and high expression in red by KM analysis, with median cutoff values. *p* values were calculated using the log-rank test). Data are expressed as the mean ± Standard Error of Mean (SEM). * *p* < 0.05 versus MCF10A cells; ** *p* < 0.01 versus MCF10A cells; **** *p* < 0.0001 versus MCF10A cells.

Increased production of lactate is the first sign of the WE, and the increase in glucose uptake becomes the fuel for glycolytic cellular metabolism [29,30,31,32,33]. To primarily verify the accumulation of lactate, we performed lactate abundance analysis on different subtypes of breast cancer using the DepMap portal. Four subtypes of breast cancer showed high lactate abundance in the metabolomics database (Figure 1A). Then, we measured the intracellular and extracellular lactate levels and glucose uptake and consumption in three breast cancer cell lines (MCF-7, T47D, and MDA-MB-231). Breast cancer cells presented with significantly higher extracellular lactate accumulation than that of normal breast epithelial cells (MCF10A), and the intracellular lactate levels were also elevated slightly (Figure 1B). Similarly, elevated glucose uptake and consumption were observed in MCF-7, T47D, and MDA-MB-231 cells (Figure 1C), which revealed that the WE is specifically prominent in breast cancer cells. These results suggested that extracellular lactate may provide a potential source of oxidizable substrate in the low-glucose microenvironment of tumors. Glucose uptake is a highly regulated event that is closely related to the proliferative state of tumor cells. Extracellular glucose is transported into cells independent of energy by facilitative-type glucose transporters (GLUTs), which are involved in glucose metabolism, the inflammatory response, and the immune response. Fourteen members of GLUTs have been identified, whose corresponding genes belong to the solute carrier 2A family *SLC2A* [34,35,36,37]. We investigated the clinical significance of the *SLC2A* family across breast cancer in the GSCA. Specifically, *SLC2A1*, *SLC2A6*, *SLC2A10*, and *SLC2A13* were significantly overexpressed, whereas *SLC2A3*, *SLC2A4*, *SLC2A9*, *SLC2A11*, and *SLC2A12* had downregulated expressions in breast tumors compared with those in normal breast epithelium (Figure 1D). According to previous results, GLUT1 is responsible for basal glucose uptake, and it is expressed in virtually all tissues in normal conditions [34,38,39]. However, GLUT6, a low-affinity glucose transporter, is widely present in the spleen, brain, and leukocytes [39,40]. *SLC2A10* has been detected in the heart, lungs, brain, liver, skeletal muscle, pancreas, placenta, and kidneys [41]. *SLC2A10* expression in cancer cell lines and tumors is unknown. *SLC2A13* is expressed primarily in the brain, with high expression in the hippocampus, hypothalamus, cerebellum, and brainstem. We then analyzed *SLC2A1* mRNA expression using BRCA (Breast Cancer) datasets and TCGA breast cancer datasets (FDR = 1.9 × 10^−11^, Figure 1E) and the GEPIA database (Figure 1F). We found that the mRNA expression levels of *SLC2A1* were significantly higher in the breast tumor. The overexpression of GLUT1 has been documented in several cancer types, including lung, brain, breast, bladder, and so on. However, mixed evidence exists in the literature regarding its expression in breast cancer. Some studies have shown that the level of GLUT1 expression correlates with specific tumor characteristics [42,43]. Therefore, we detected GLUT1 expression in different breast cancer cell lines. The protein expression of GLUT1 showed a higher expression in MCF-7, MDA-MB-231, and inflammatory breast cancer cells than it did in non-tumorigenic epithelial MCF10A cells (Figure 1G). The experiment results indicated that the MCF-7 cell model overexpressed GLUT1 stably and was stable in vitro. Consistent with these findings, our results have shown that GLUT1, the major glucose transporter in mammalian cells, can be a potential target for developing therapeutic strategies to target breast tumor glycolysis. We then analyzed the relationship between *SLC2A1* expression and survival outcomes in patients with breast cancer. We found that patients with *SLC2A1* overexpression exhibited a significantly unfavorable prognosis both in terms of OS (Figure 1H, HR = 1.29, logrank *p* = 0.0099) and DMFS (HR = 1.26, logrank *p* = 0.0035), which suggested that the overexpression of *SLC2A1* is positively correlated with a worse overall survival rate in the glucose metabolism pathway. This can be a potential clinical therapeutic target for decreasing the excessive amount of glucose uptake and readjusting abnormal glycometabolism in patients with breast cancer during chemotherapy.

### 2.2. OBHS Decreased GLUT1 Expression Requires PI3K/Akt Signaling Pathway in MCF-7 Cells

**Figure 2:**
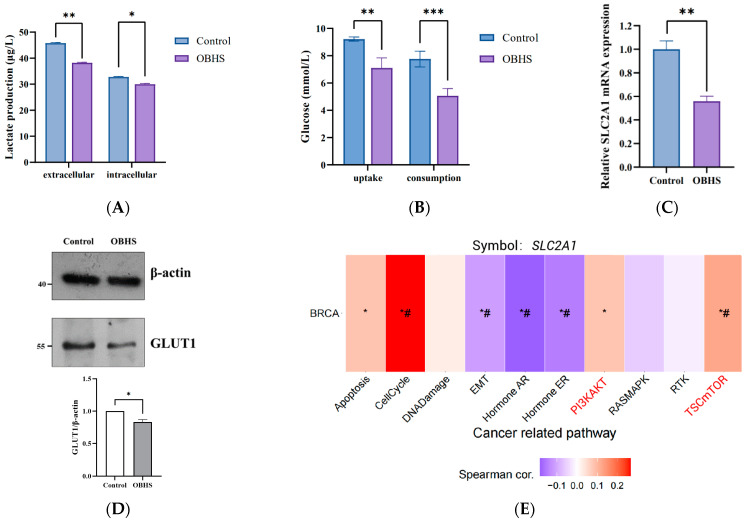

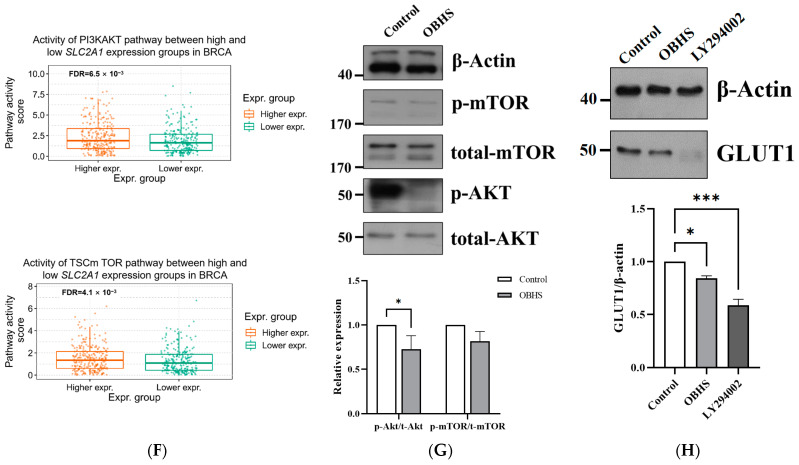
OBHS significantly inhibited glucometabolic via PI3K/Akt-GLUT1 pathway. MCF-7 cells were seeded at a density of 10^6^ cells/well and treated with OBHS for 24 h. (**A**) Lactate concentration and (**B**) glucose uptake and glucose consumption were decreased by OBHS (10 μM, purple bars) in MCF-7 cells. (**C**) *SLC2A1* mRNA expression was downregulated in 5 h OBHS-treated MCF-7 cells. (**D**) Protein levels of GLUT1 were decreased by OBHS for 24 h treatment. (**E**) GSCA summarized the percentage of breast cancer cells in which the mRNA expression level of the *SLC2A1* gene had a potential effect on pathway activity. The activated signaling pathway is shown in the red font. * *p* ≤ 0.05 versus low *SLC2A1* expression groups; # FDR ≤ 0.05 versus low *SLC2A1* expression groups. (**F**) Activities of PI3K/Akt and mTOR pathways between high- and low-*SLC2A1* expression groups in BRCA. (**G**,**H**) Immunoblot of signaling-pathway-related gene expression using the indicated antibodies in MCF-7 cells treated with OBHS and LY294002. Data are expressed as the mean ± SEM. * *p* < 0.05 versus control; ** *p* < 0.01 versus control; *** *p* < 0.001 versus control.

An increased aerobic glycolysis rate is a common metabolic feature in malignant tumors. Our data indicated that MCF-7 cell line stably expresses GLUT1, making it an extraordinarily active metabolic system. To test whether OBHS has the suppressed effect on WE, a lactate concentration test was performed at room temperature with the L-Lactate Assay Kit. The data demonstrated that OBHS treatment decreased both intracellular and extracellular levels of lactate (Figure 2A). Reducing glucose uptake and blocking its transport pathway are vital steps for small-molecule compounds to inhibit the growth of cells [44]. MCF-7 cells were cultured in phenol red-free medium with a high (25 mM) concentration of D-glucose to simulate the hyperglycemia tumor tissue environment for 2 days, followed by treatment with a low concentration of OBHS to determine how OBHS affected the abnormal glucose flux in BRCA cells. As expected, the 10 μM OBHS treatment presented the apparent inhibitory effect in terms of both glucose uptake and consumption (Figure 2B), indicating a possible inhibitory function of OBHS toward the glycolytic pathway. We then performed a time–course and concentration–course analysis of *SLC2A1* gene expression to determine the reason for decreasing glucose uptake following the OBHS treatment of MCF-7 cells. The mRNA expression level of *SLC2A1* was significantly decreased by OBHS within 5 h of exposure, as detected by RT-qPCR (Figure 2C). GLUT1 protein levels were found to be downregulated at a low OBHS concentration (Figure 2D). Taken together, these data indicated that OBHS reprogrammed GLUT1 to inhibit aerobic glycolysis in breast cancer cells.

The Akt and mTOR signaling pathways are important regulators of glucometabolic homeostasis, which directly stimulates glucose consumption and enhances glycolysis [45,46,47,48,49,50,51]. To investigate their regulatory relationships, we summarized the capacity for which *SLC2A1* mRNA expression has a potential effect on pathway activity in breast cancer. The results showed that *SLC2A1* had a stronger of activation effect on PI3K/Akt and mTOR pathways (Figure 2E). The higher mRNA expression level of *SLC2A1* had greater activation on the PI3K/Akt (FDR = 6.5 × 10^−3^) and mTOR (Figure 2F, FDR = 4.1 × 10^−3^) signaling pathways. Previous studies showed that the expression rates of p-Akt and GLUT1 were higher in breast cancer and DCIS than they were in normal tissue [52]. High-level Akt activity in cancer cells is reflected by their high-level glucose uptake activity and GLUT1 expression [53,54,55]. Moreover, Akt controls the plasma membrane localization of GLUTs, and Akt can regulate GLUTs plasma membrane expression [56,57]. mTOR regulates glycolysis via the transcription and protein synthesis of GLUTs and glycolytic enzymes [58]. To determine if OBHS’s ability to induce the inactivation of the Akt/mTOR pathway in MCF-7 cells is correlated with its inhibition of GLUT1, we performed Western blot analysis, which showed the downregulated expression of p-Akt after OBHS treatment, and unfortunately, there was no significance in mTOR phosphorylation (Figure 2G). Five µM of LY294002 leads to the obvious inhibition of GLUT1 (Figure 2H). Overall, these data suggested that OBHS can play an important role in disrupting the axis of PI3K/Akt-GLUT1, which results in metabolic reprogramming in breast cancer cells.

### 2.3. OBHS Suppressed Energy Metabolism by Altering the Expression Levels of Associated Metabolic Intermediates

**Figure 3:**
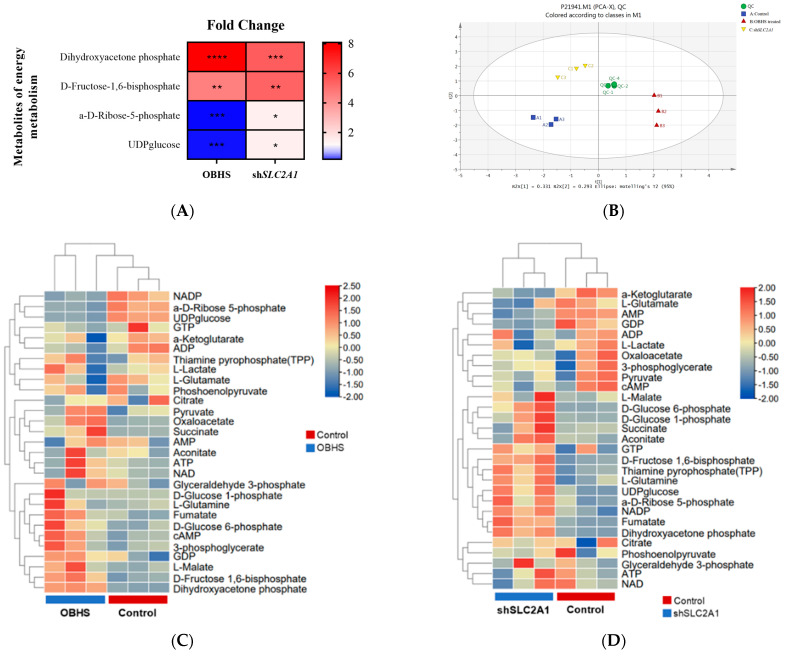

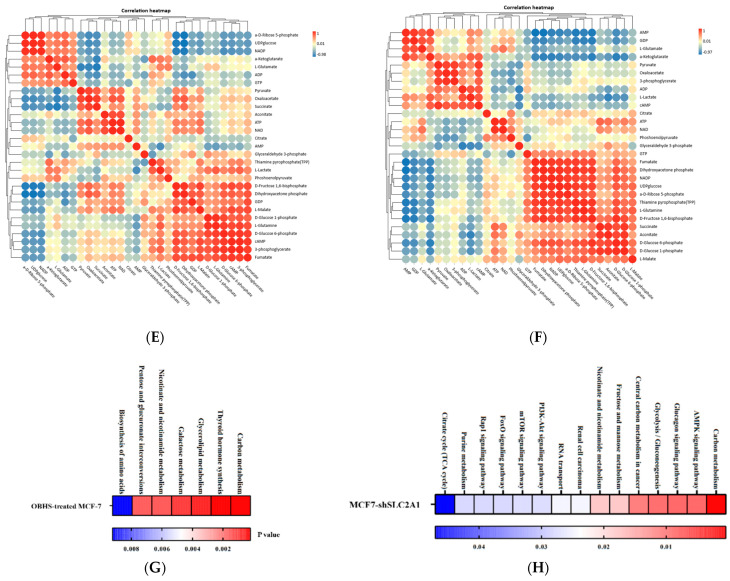
OBHS was crucial for the GLUT1-dependent reprogramming mechanism in the energy metabolism pathway. The *y*-axis indicates the metabolites, and the *x*-axis indicates the fold change of the metabolites in (**A**) OBHS-treated (red triangles) and *SLC2A1* knockdown (yellow triangles) MCF-7 cells. (**B**) PCA score plots of OBHS-treated (red triangles) and *SLC2A1* knockdown (yellow triangles) MCF-7 cells samples. Heat map and hierarchical clustering of metabolites in (**C**) OBHS-treated and (**D**) MCF7-sh*SLC2A1* cells and controls. Fold change > 0 represents upregulated metabolites, which are shown in red. Fold change < 0 represents downregulated metabolites, which are shown in blue. Heat map of the correlation coefficient matrix shows the ratio of correlation coefficients of 40 metabolites from the data in (**E**) OBHS-treated and (**F**) sh*SLC2A* cells. Pearson R is between −1 and +1. The correlation coefficient R between metabolites is represented by color and circles, where R > 0 represents a positive correlation and is represented in red. R < 0 represents negative correlation, which is shown in blue. Heat maps generated from the targeted metabolomics array depicting *p* values for the most highly up- and downregulated metabolites following (**G**) OBHS treatment or (**H**) *SLC2A1* knockdown. * *p* < 0.05 versus control; ** *p* < 0.01 versus control; *** *p* < 0.001 versus control; **** *p* < 0.0001 versus control.

Next, we attempted to perform targeted metabolomics analysis in non-treated, OBHS-treated, and sh*SLC2A1* MCF-7 cells to evaluate the measurable systemic changes with potential diagnostic significance for the early phase of metastasis development. To determine the concentration of selected metabolites from studied pathways, three liquid chromatography–mass spectrometry-targeted metabolomics-based methods were applied. We used liquid chromatography–mass spectrometry to quantitate 40 metabolites from the glycolytic pathway, tricarboxylic acid cycle (TCA), pentose phosphate pathway (PPP), oxidative phosphorylation pathway, and other pathways. Among the forty metabolites successfully quantified, four metabolites were differentially abundant both in OBHS-treated MCF-7 (*p* < 0.001) and MCF7-*shSLC2A1* cells (*p* < 0.05) in the control group (Figure 3A). Principal component analysis (PCA) reflected distinct patterns in all the cells analyzed (Figure 3B). Heatmap analysis of the metabolites identified the same and different effects between OBHS-treated and sh*SLC2A1* MCF-7 cells (Figure 3C,D). The changes in cellular metabolism were shown to be closely associated with cancer progression. Furthermore, glucose phosphorylation caused transfection between D-fructose-1,6-bisphosphate and glyceraldehyde-3-phosphate; consequently, D-fructose-1,6-bisphosphate accumulated in the cells. The only difference was that OBHS directly inhibited the phosphorylation of D-glucose-6-phosphate, but the shRNA knockdown of *SLC2A1* stressfully promoted glycogen metabolism to compensate for glucose metabolism. We suspected that OBHS has a synthetic effect on glucose metabolism reprogramming, including a GLUT1-based specific influence. Based on these findings, we constructed a heatmap of the metabolite correlation coefficient matrix consisting of the ratio of the correlation coefficients of 40 metabolites from the data. We further confirmed that OBHS inhibited PPP by blocking the formation of intermediate products in the glycolytic pathway and TCA (Figure 3E). In addition, the database showed that the suppression of the glycolytic pathway and TCA in MCF7-sh*SLC2A1* cells induced a temporarily increased level of glycogen metabolites (Figure 3F). We also statistically analyzed the effects of OBHS treatment on universally enriched metabolites in the signaling pathway, and these data confirmed that the OBHS treatment of MCF-7 cells significantly altered the expression of multiple metabolites in the carbon metabolism pathway. Similar results were observed in MCF7-sh*SLC2A1* cells, which showed that metabolites in the carbon metabolism pathway were among the top five most induced metabolites (Figure 3G,H).

### 2.4. Anti-Glucometabolic Effect of OBHS in MCF-7 Cells Occurred via the Reprogramming of the Glycolytic Metabolism Pathway in Breast Cancer Cells

**Figure 4:**
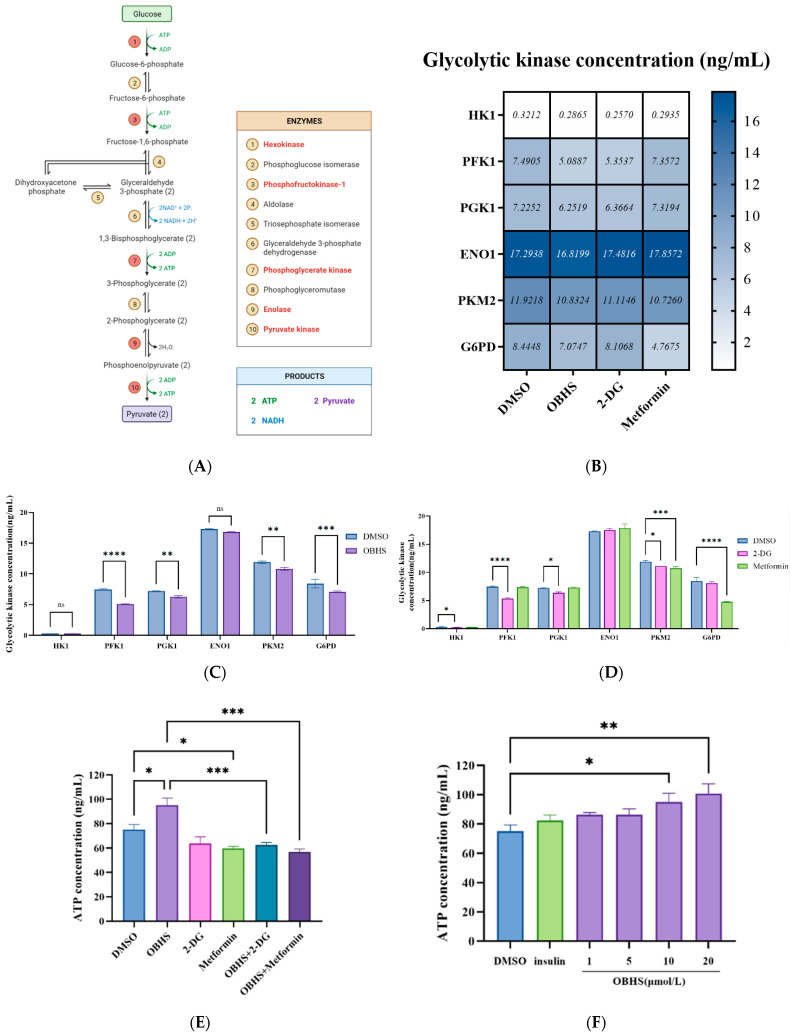
OBHS regulates glucose and oxidative phosphorylation-related enzyme concentration among glycolytic and PPP. (**A**) A schematic diagram of the glucose metabolic flux. (**B**) Heat map depicting the effects of OBHS, 2-DG, and metformin on the enzymes concentration. The mean value were marked in each cell. The cells were treated with (**C**) OBHS (purple bars), (**D**) 2-DG (pink bars), and metformin (light green bars) for 24 h, and the supernatant of cell lysates was collected after repeated freezing and thawing of cells for lysis to measure intracellular concentrations of HK1, PFK1, PGM1, ENO1, PKM2, and G6PD. The concentrations were analyzed using corresponding ELISA assay kits. The cells were treated by (**E**) 10 μM OBHS, 12.5 μM 2-DG, and 8 μM metformin compound and combination for 24 h, and we collected the supernatant of cell lysate to measure in MCF-7 cells. (**F**) MCF-7 cells were given incremental levels of OBHS. The concentration was analyzed by corresponding ELISA assay kit. Data are expressed as the mean ± SEM. ns: *p* > 0.05; * *p* < 0.05 versus DMSO; ** *p* < 0.01 versus DMSO; *** *p* < 0.001 versus DMSO; **** *p* < 0.0001 versus DMSO.

Next, we sought to determine the potential biological effects of OBHS in breast cancer cells. An increase in the activity of rate limiting enzymes systems involved in glycolysis and shifting metabolic patterns revealed significant changes in multiple tumor cells [59,60]. Hence, to explore whether there was a further depressive effect on glucose digestion, the production of key enzymes (Figure 4A) was examined. In addition, we sought to compare the potential biological effects of OBHS and the antidiabetic agents, 2-DG and metformin. The experiments were repeated under the same conditions following the administration of different drugs and different concentrations. Using ELISA, we measured the concentrations of enzymes involved in the WE, including HK1, PFK1, PGK1, ENO1, and PKM2 (Figure 4B). OBHS-treated cells exhibited lower concentrations of PFK1, PGK1, and PKM2, but not of HK1 and ENO1. Specifically, 2-DG-treated cells altered the concentrations of HK1, PFK1, PGK1, and PKM2. Metformin treatment led to a decrease in the level of rate-limiting enzymes in the final step of glycolysis, PKM2, in MCF-7 cells (Figure 4C,D). Of note, HK1 and PFK1 played central roles in the regulation of glycolysis by catalyzing the MgATP-dependent phosphorylation of fructose-6-phosphate, forming ADP and D-fructose-1,6-bisphosphate, which are considered to be pacemakers of glycolytic flux [61]. OBHS demonstrated a better inhibiting effect than 2-DG did, indicating that OBHS may suppress glucose phosphorylation, leading to limited energy flux and disturbing the balance between anabolism and oxidation reduction reactions. Furthermore, a significantly lower concentration of PKM2 was observed in OBHS-treated cells than in those in the control group, which suggested that OBHS has an effect on oxidative phosphorylation [62,63]. These results suggest that OBHS-affected biotargets are involved in both glucose phosphorylation and oxidative phosphorylation in glycolysis; the results for 2-DG and metformin were different from those for OBHS.

Next, we explored the biological effects of OBHS in oxidative phosphorylation. We considered whether OBHS affects PPP, a bypass of glycolysis, via the regulation of G6PD as a key process in carcinoma proliferation. PPP begins with glucose-6-phosphate, and glyceraldehyde-3-phosphate and fructose-6-phosphate are generated in the final step. Researchers have found that PPP plays an important role in tumor proliferation, apoptosis, invasion, and drug resistance. Thus, we monitored the change in OBHS-mediated G6PD production and activity in vitro to test the effects on PPP. ELISA showed that the G6PD concentration was markedly decreased by the OBHS treatment (Figure 4C), resulting in the subsequent activation of glucose-6-phosphate oxidization and the probable generation of ATP. Meanwhile, an increase in ATP production was observed when MCF-7 cells were treated with high concentrations of OBHS (Figure 4F). In addition, 120 ng/mL insulin dramatically stimulated the progress of cell glucose uptake and consumption. These data provided a support to define it as a negative control and the basis for the subsequent establishment of the hyperinsulinemia tumor cells model. However, insulin has no effect on intracellular ATP production. It is worth noting that hyperinsulinemia may not affect ATP generation. ATP production hit a plateau with a low concentration of OBHS, and an apparent additional effect was detected with a high concentration of OBHS. Surprisingly, the exceptional increase was consistent with the aforementioned results of PKM2-induced activated PPP. Although OBHS had a negative effect on intracellular energy production, we unexpectedly discovered that the combination of OBHS and 2-DG or metformin showed to no significant difference when it was compared with OBHS alone (Figure 4E). These findings shed light on how the reprogramming effect on energy metabolism by OBHS can be enhanced in breast cancer cells. Our findings showed that blocking lactate excretion reversely regulated the conversion of pyruvate to lactate catalyzed by lactate dehydrogenase 1 (LDH1), and PKM2 downregulation was associated with increased metabolic intermediates. Meanwhile, both the downregulation of PFK1 and decreased activation of PPP resulted in an increased concentration of dihydroxyacetone phosphate and D-fructose-1,6-bisphosphate. Consistent with these findings, this functional overlap prompted us to hypothesize that an increase in metabolic intermediates and pyruvate aggravated the oxidative phosphorylation of glucose, which may generate primarily ATP-based energy.

### 2.5. Enhanced ROS Fluxes Imply Mitochondria Toxicity by OBHS and Lactate-Inducted Cell Apoptosis

**Figure 5:**
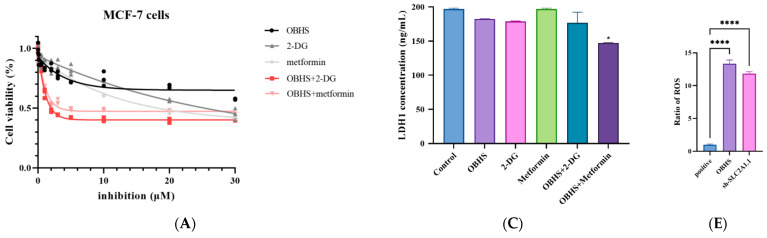

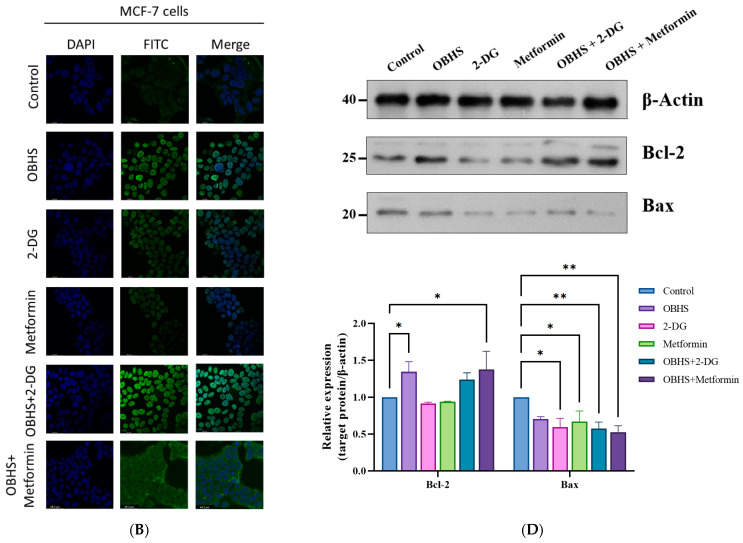
OBHS promoted lactate-induced cell apoptosis caused by the increased ratio of ROS in MCF-7 cells. (**A**) OBHS led to a reduction in cell viability in MCF-7 cells. (**B**) Representative images of TUNEL staining. TUNEL-positive apoptotic cells were shown in green and DAPI nuclear staining was showed in blue (scale bar = 46.2 μm). (**C**) The cells were treated with 15 μM OBHS, 10 μM 2-DG (pink bars), 10 μM metformin (green bars), a combination of metformin and OBHS (dark purple bars), or 2-DG and OBHS (lake blue bars) for 24 h, and we collected the supernatant of cell lysate by repeated freezing and thawing lyses the cells to measure LDH1 concentration. (**D**) OBHS treatment and the combinations promoted apoptosis in MCF-7 cells. (**E**) Both OBHS treatment and *SLC2A1* knockdown led to an increased ROS level in MCF-7 cells. Data are expressed as the mean ± SEM. n.s., not significant. *p* values are indicated. * *p* < 0.05, ** *p* < 0.01, and **** *p* < 0.0001.

We first assessed cell viability using a CCK-8 assay to investigate whether the lactate secretion-inhibitory effect of these three compounds was associated with the induction of apoptotic cell death. In addition, we further determined whether the combination with antidiabetic agents had a synergistic effect. MCF-7 cells were more sensitive to the combination, which showed the powerful suppression of proliferation, implying that OBHS and two antidiabetic agents might aim at various biotargets in the WE (Figure 5A). A few TUNEL-positive cells were observed in the control group. Compared with the control group, the number of TUNEL-positive cells in the treated group increased significantly. The OBHS-treated group and the combination significantly increased the number of TUNEL-positive cells in MCF-7 cells (Figure 5B). It was suggested that OBHS could accelerate cell apoptosis. Then, we examined LDH1, a key rate-limiting factor for the lactate generation process, and the production concentration in MCF-7. Intracellular LDH1 was decreased upon the addition of the combination (Figure 5C). We hypothesized that the combination of OBHS and metformin might be more inclined to function during lactate formation. On the contrary, OBHS resulted in a greater inhibition effect on intracellular-to-extracellular transportation than the other one did. To confirm the apoptosis-promoting effect of the OBHS treatment and the combinations, Bcl-2 and Bax, mitochondrial-apoptosis-related proteins, were detected, the levels of which were significantly increased and decreased, respectively, (Figure 5D). When the energy needed for tumor cell proliferation comes from oxidative scale acidification, ROS production occurs, and the accumulation of ROS can activate cell apoptosis [64]. To further confirm the above-mentioned suppositions, we also set out to analyze the intracellular reactive oxygen species (ROS) level in MCF-7 cells. We suggested that OBHS was linked to enhance mitochondrial superoxide production using an oxidative phosphorylation mechanism to generate ROS and promoted apoptotic cell death (Figure 5E). Taken together, we reasoned that OBHS palpably reduces extracellular lactate build-up, resulting in an abnormal cytoplasmic growth environment that was not fit for the growth of the cancer cells, and ultimately, promoted cell apoptosis. Its mechanism may be related to the increase in the intracellular ROS level.

### 2.6. OBHS Had Suppression Effect on Breast Cancer Progression and Proliferation, and It Reprogrammed the Metabolic Pathways In Vivo

**Figure 6:**
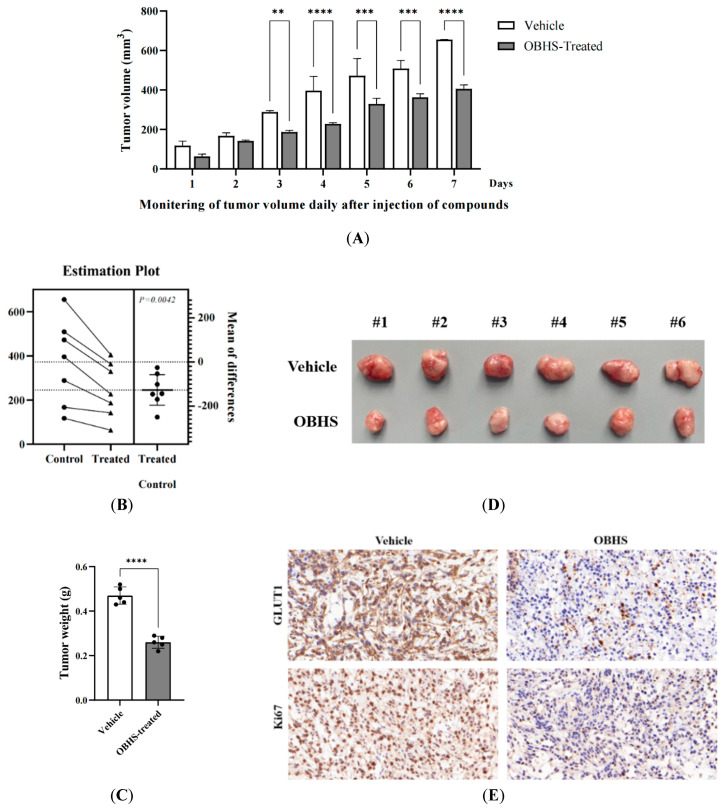
OBHS significantly delayed disease progression in the MCF-7 breast cancer mouse model. (**A**) OBHS suppressed the tumor growth in the MCF-7 tumor mouse model. The average tumor sizes of the vehicle (*n* = 6) and OBHS-treated groups (*n* = 6) were plotted after inoculation. Data are represented as mean ± SD, and the statistical analysis between vehicle and drug-treated groups was performed by a two-way ANOVA with Tukey’s test and (**B**) paired-sample *t* test. *p* values are indicated. ** *p* < 0.01, *** *p* < 0.001, and **** *p* < 0.0001. (**C**) OBHS-treated MCF-7 cells had slower growing and smaller tumors. (**D**) Representative tumor sizes after seven rounds of drug treatment. (**E**) IHC for Ki67 and GLUT1 in subcutaneous xenograft tumors. Scale bars, 20 μm.

We established xenograft tumors by subcutaneously injecting MCF-7 cells into male athymic mice to examine the role of OBHS treatment in breast cancer progression. The mice were randomized to OBHS until the tumors reached an approximate volume of 100 mm^3^. The treatment with OBHS for 1 week led to a significant decrease in the tumor volume compared with that in the control mice (Figure 6A). The difference between the OBHS- and vehicle-treated groups was significant (*p* = 0.0042) (Figure 6B). The OBHS treatment effectively suppressed the growth of xenograft tumors (Figure 6C,D). We then evaluated cell proliferation using the nuclear protein Ki67 and glucose transporter (GLUT1) activity using the immunohistochemistry analysis of tumor sections. We found that the OBHS treatment resulted in a reduced number of Ki67+- and GLUT1-positive tumor cells, which showed anti-proliferative and anti-glycolytic activities (Figure 6E). These findings led to the exploration of the metabolic characteristics associated with OBHS.

## 3. Discussion

**Figure 7:**
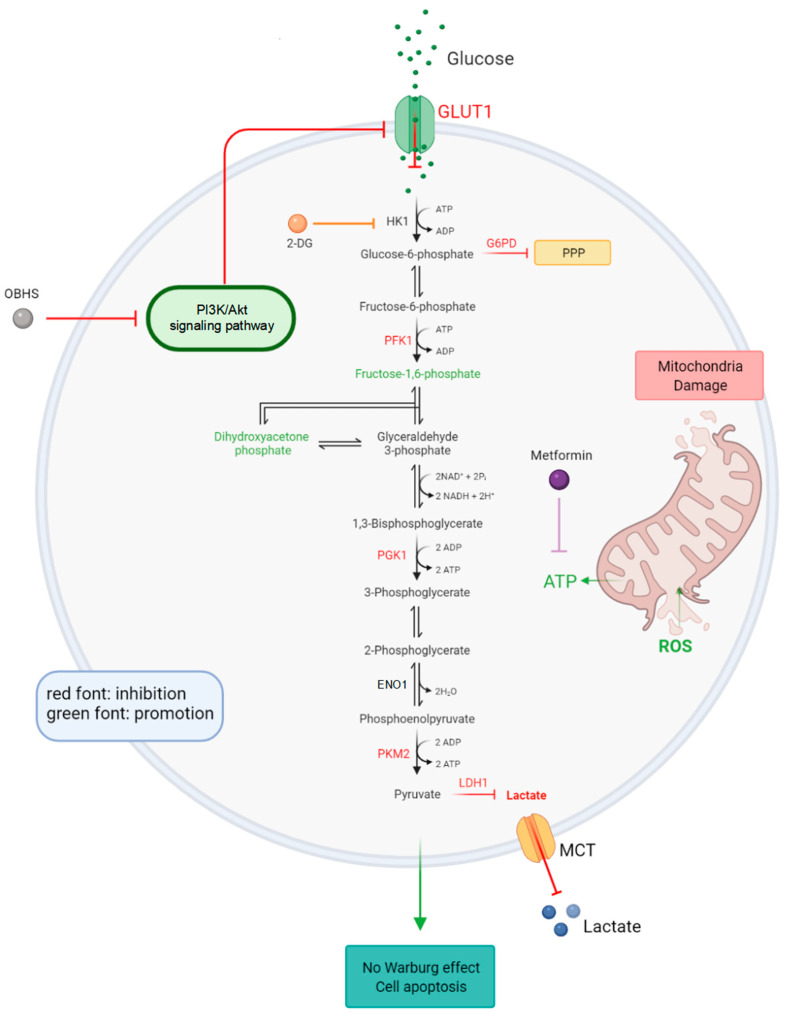
Proposed mechanism of which OBHS remodels abnormal glycometabolism, referring the experiment results. The inhibited pathway or reaction is shown in the red font, and the promoted pathway or reaction is shown in the green font.

Previous studies have demonstrated that breast cancer cells have substantially different glycometabolisms compared with those of normal cells or tissues, which exhibit upregulation in glycolysis, glycogen metabolism, and gluconeogenesis [65]. Energy supply plays a critical role in tumor cell proliferation and metastasis [66,67,68], but the impact of OBHS on this has not been studied. In the present study, we sought to characterize the OBHS-targeted tumorous glucometabolic state in breast cancer and to elucidate the remodeling mechanisms through which OBHS functions in this disease (Figure 7).

Increased aerobic lactate production and excess glucose uptake are reversed in breast cancer cells compared with those activities in normal mammary cells. We found that GLUT1 is highly associated with glycometabolism in breast cancer cell lines, which is consistent with previous findings [45,69,70,71,72,73]. Our results suggest that patients with decreased *SLC2A1* expression have a decreased OS and relapse-free survival rates for luminal A subtypes of breast cancer. OBHS caused a decrease in both lactate production and glucose flux. Glucose flux was shown to be blocked in GLUT1-downregulated MCF-7 cells, which resulted in low glycolytic activity in breast cancer cells. Here, we characterize the critical role of OBHS, a novel SERM, in the regulation of the glucose concentration in the tumor microenvironment by specifically downregulating the expression of GLUT1 to limit cellular energy intake. Lactate can be classified into intracellular and extracellular lactate, which are associated with intracellular and extracellular transportation and initial formation, respectively. Previous experiments measured FDG uptake in the core and periphery of the tumor and found that increased lactic acid accumulation suppresses FDG uptake in a dose-dependent manner. The disadvantageous microenvironmental factors for tumor cell growth, such as low acidity (decreased extracellular lactate level) and decreased nutrient supply (decreased glucose uptake and consumption), which influence cell biology include cell viability, proliferation, and metabolism after the use of energy. According to the database analysis in GSCA, we explored the upstream pathway of GLUT1, which is the PI3K/Akt/mTOR pathway, and found that OBHS inhibits the phosphorylation activity of Akt, suggesting that the PI3K/Akt-GLUT1 axis is critical for glycolysis remodeling. These findings have been supported by previous results showing that the phosphorylation of Akt promotes the glucose metabolism of cancer cells by increasing the expression of GLUT1 and regulating key glycolytic enzymes simultaneously [68,74,75]. Particularly, Akt stabilized GLUT1 at the plasma membrane (PM) by inhibiting its endocytosis, increasing aerobic glycolysis [76,77]. Multiple reports, the inhibition of the PI3K/mTOR pathway effectively suppressed the membrane localization of facilitative GLUT1 [78,79,80,81]. Conversely, GLUT1 was released from the early endosome to the PM when PI3K/mTOR inhibition was stopped.

Mechanistically, we revealed that OBHS inhibited glycolysis in step six of the reaction. The main indication was the slight increase in dihydroxyacetone phosphate resulting from the hindered transfection from glyceraldehyde-3-phosphate to 1,3-bisphosphoglycerate. The effects of OBHS treatment on glycolysis in cancer are striking in so far as the treatment partially, directly, or indirectly disrupted the glucose metabolism, dramatically reduced metastasis, and induced MCF-7 cell apoptosis. Here, we found that sh*SLC2A1* unexpectedly enhanced glycogenesis, which is thought to adapt abnormal glucose levels to sustain metabolism for cell survival. Meanwhile, OBHS functions in the remodeling of metabolic pathways, not just by reducing GLUT1 expression. Thus, our findings across targeted metabolic analyses provide a strong rationale for the potential use of a metabolic inhibitor.

We further found that OBHS can rapidly regulate different kinases, not only those related to glucose phosphorylation, but also those related to oxidative phosphorylation in glycolysis. The dramatic inhibition-inducted increased activity of oxidative phosphorylation should be further studied. Here, we made a reasonable conjecture that the uncontrollable inhibition of PKM2 expression enabled glycolytic intermediates to enter PPP. The disturbed balance between pyruvate synthetic pathways and the oxidation reduction pathway may contribute to abnormal ATP synthesis.

The degree of downregulation of LDH1 expression has been used to define the lactate status. Combination therapy of OBHS and 2-DG or metformin had a greater inhibitory effect on lactate synthesis, as revealed by the decrease in LDH1 expression. These results imply that the OBHS treatment alone affected the intracellular-to-extracellular transport process, whereas combination therapy affected intracellular synthesis rather than transportation. Furthermore, the LDH1 inhibitor was sufficient to suppress the WE by promoting the oxidative phosphorylation of glucose metabolism, which resulted in NADH oxidization-inducted tumor cell growth arrest [14]. Increased intracellular lactate build-up results in a decreased intracellular pH value and an elevated pyruvate level, which both induce apoptosis in breast cells. Furthermore, OBHS exerts synthetic lethal effects with 2-DG and delays tumor progression in vitro. The explanations for this successful combination strategy may be due to different complementary biotargets of the glycolysis pathway, which enhance the cells’ sensitivity to 2-DG. Given the clinical data and the profound effects of OBHS with 2-DG or metformin in breast cancer cells, we present an optimistic view that OBHS and its conjugates could be potential anti-tumor glycolysis candidates for breast cancer patients in combination with other antidiabetic agents, replacing the clinical monotherapy strategy. In MCF-7 cells, we observed a significant increase in ROS production when they were treated with low concentration of OBHS. In addition, we observed that the OBHS-induced elevation of ROS was associated with mitochondrial damage. Previous studies have shown that mitochondrial structure damage is related to cell apoptosis [82,83,84,85,86]. In vivo experiments of OBHS using an endometriosis mouse model demonstrated that OBHS suppresses tumor growth. Our findings suggest that OBHS functions as an antitumor agent by suppressing GLUT1 expression and cellular proliferation.

Collectively, our results highlight a clinically feasible scenario to use OBHS as an anticancer drug and glycometabolism regulator for targeted therapy. To the best of our knowledge, this is the first study to identify OBHS as a regulator of energy metabolism in cancer. Therefore, it will be important to further identify the potential clinical significance of OBHS, as well as to discover new therapeutic targets for breast cancer.

## 4. Materials and Methods

### 4.1. Chemicals and Reagents

OBHS was designed and synthesized by our team, and the synthesis process has been described in previous publications [17]. Metformin (98%, Reagent grade) and 2-DG (98%, Reagent grade) were purchased from Yuanye Bio-Technology Co., Ltd. (Shanghai, China), dissolved in dimethylsulfoxide (DMSO), and then stored at −80 °C. Insulin was purchased from Sigma. Dulbecco’s modified Eagle medium (DMEM), low glucose DMEM, Roswell Park Memorial Institute (RPMI) 1640, L15 (Leibovitz’s), fetal bovine serum (FBS), trypsin, and penicillin/streptomycin were obtained from Gibco (New York, NY, USA). DMEM/F-12 with no phenol red was purchased from HyClone Proteins (Logan, UT, USA). Hieff Trans Liposomal Transfection Reagent and DH5α chemically competent cells were purchased from Yeasen Biotechnology (Shanghai, China).

### 4.2. Cell Culture

MCF-7 and MDA-MB-231 human breast cancer cells were cultured in DMEM supplemented with or without a high concentration of D-glucose (4500 mg/L) containing 10% FBS, 2 mM glutamine, and 100 units/mL penicillin/streptomycin (Gibco, New York, NY, USA). T47D human ductal breast cancer cells were cultured in RPMI 1640 containing 10% FBS, 0.2 U/mL insulin, and 100 units/mL penicillin/streptomycin (Gibco, New York, NY, USA). MCF10A human normal mammary epithelial cells were cultured in DMEM/F-12 supplemented with 5% horse serum, 20 ng/mL epidermal growth factor, 0.5 μg/mL hydrocortisone, 10 μg/mL insulin, 1% non-essential amino acid, and 1% penicillin/streptomycin (P/S). MDA231-LM2 human inflammatory breast cancer cells were cultured in L15 supplemented with 10% FBS and 100 units/mL penicillin/streptomycin (Gibco, New York, NY, USA). MCF-7, T47D, MDA-MB-231, and MCF10A cells were kindly provided by Procell Life Science and Technology Co., Ltd. (Wuhan, China). MDA231-LM2 cells were kindly provided by Otwo Biotech Co., Ltd. (Shenzhen, China). Of note, 48 h before the experiments, the cells were switched to phenol red-free DMEM supplemented with 5% charcoal-stripped FBS, 2 mM glutamine, and 100 units/mL penicillin/streptomycin. These cells were maintained in a humidified atmosphere of 5% CO_2_ at 37 °C.

### 4.3. Cell Viability Studies

The cells were seeded into 96-well plates at a density of 5000 cells per well. Subsequently, the cells were treated with different concentrations of OBHS, 2-DG, metformin, and the combination on the next day and incubated for 48 h. Then, 10 μL of Cell Counting Kit-8 (CCK-8) solution was added to each well (100 μL/well), and the plates were incubated at 37 °C for 45 min following the manufacturer’s protocol. The absorbance was measured at 450 nm using a microplate reader (Bio-Tek Instruments, Winooski, VT, USA). All experiments were conducted in triplicate.

### 4.4. Survival Analysis

The retrospective analysis of specific gene expression and patient survival was conducted using the online Kaplan–Meier plotter for breast cancer survival analysis (https://kmplot.com/analysis/, accessed on 4 April 2023). Deficiency in the available patient follow-up data precluded the analysis of overall survival (OS) and distant metastasis-free survival (DMFS) in breast cancer. The hazard ratio (HR) with 95% confidence intervals and the log-rank *p* values are indicated.

### 4.5. Western Blot Analysis

Cells were lysed using RIPA lysis buffer (Biosharp, Beijing, China) with a protease inhibitor cocktail (Cell Signaling Technology, Boston, MA, USA). Total soluble protein was electrophoresed from 8% to 12% on sodium dodecyl sulfate (SDS)-polyacrylamide gels and transferred onto a polyvinylidene fluoride (PVDF) membrane (Millipore, Burlington, MA, USA). After blocking with 5% albumin from bovine serum (BSA) or skimmed milk for 1 h at room temperature, the blots were probed with primary antibodies to GLUT1 (dilution 1:1000, ABclonal, Wuhan, China), B-cell lymphoma-2 (Bcl-2, dilution 1:5000, Proteintech, Rosemont, IL, USA), BCL2-Associated X (Bax, dilution 1:1000, Proteintech, Rosemont, IL, USA), p-Akt (Ser473), total Akt (dilution 1:1000, ABclonal, Wuhan, China), p-mTOR (Ser2448) or total mTOR (dilution 1:1000, Santacruz Biotechnology, Dallas, TX, USA) and kept overnight at 4 °C, followed by incubation with horseradish peroxidase (HRP)-labeled secondary antibodies (Biofly, Chengdu, China) for 1 h at room temperature. After washing 3 times with 1% TBST, the bands were visualized using an efficient chemiluminescence (ECL) kit (Millipore, Burlington, MA, USA). β-actin (Sigma, Saint Louis, MO, USA) was used as the loading control.

### 4.6. Real-time Quantitative PCR (RT-qPCR)

Total cellular RNA was isolated from MCF-7 cells using an RNA extraction kit (Cwbio, Taizhou, China) according to the manufacturer’s instructions. Of noted, 2 μg of total RNA was used for reverse transcription with M-MLV reverse transcriptase (Promega, Madison, WI, USA). RT-qPCR (CFX Connect Real-Time PCR, Bio-Rad, Hercules, CA, USA) was performed using a ChamQ™ Universal SYBR qPCR Master Mix (MedChemExpress, Shanghai, China). PCR was performed with an initial denaturation at 95 °C for 5 min, followed by 35 cycles of annealing at 95 °C for 15 s and extension at 60 °C for 45 s. All primers used for real-time PCR analysis were synthesized by Tianyi Huiyuan, Beijing, China. Glyceraldehyde-3-phosphate dehydrogenase (GAPDH) was used as the internal control.

Sequences of primers:

*GAPDH* forward primer: GGACCTGACCTGCCGTCTAG;

*GAPDH* reverse primer: GTAGCCCAGGATGCCCTTGA;

*SLC2A1* forward primer: AAGCTGACGGGTCGCCTCATG;

*SLC2A1* reverse primer: CTCTCCCCATAGCGGTGGACC;

*SLC2A10* forward primer: CCCAGTTGAAGCTGTTGCAG;

*SLC2A10* reverse primer: CTGCAACAGCTTCAACTGGG.

### 4.7. Glucose Consumption and Uptake Assay

Approximately 1 × 10^6^ cells per well in a six-well plate were treated with or without OBHS for 24 h. The culture medium and cell lysate supernatant were collected to measure extracellular and intracellular glucose concentrations, respectively. First, the culture medium was collected, and the cells were washed twice with cold phosphate-buffered saline (PBS) before lysis using 1.5% TritonX-100 (Beyotime, Shanghai, China) for 40 min without centrifugation to release intracellular glucose. Glucose consumption and uptake were measured via the glucose oxidase–peroxidase method using a Glucose Assay Kit (Rsbio, Shanghai, China) according to the manufacturer’s instructions. The results represent six independent experiments. The optical density (OD) value in each well was detected at a wavelength of 505 nm using a microplate reader (Bio-Tek, Winooski, VT, USA). The concentration was calculated according to the formula listed below:

(1) $$\mathrm{Glucose}\left(\mathrm{mmol}\cdot L^{-1}\right)=\frac{{\mathrm{OD}}_{\mathrm{sample}}\;\mathrm{value}-{\mathrm{OD}}_{\mathrm{control}}\;\mathrm{value}}{{\mathrm{OD}}_{\mathrm{standard}}\;\mathrm{value}-{\mathrm{OD}}_{\mathrm{control}}\;\mathrm{value}}\times \mathrm{standard}\left(5.55\;\mathrm{mmol}\cdot L^{-1}\right)$$

### 4.8. Quantification of Enzymatic Production

Approximately 1 × 10^6^ cells per well in a six-well plate were treated with or without OBHS for 24 h at 37 °C. The cells were then collected and washed twice with cold PBS, followed by repeated freezing and thawing for lysis. Next, the lysed cells were centrifuged at 3000× *g* for 5 min at 4 °C, and the supernatant was collected. The cell lysate was stored at −80 °C before analysis. Enzymatic production in cell lysates was determined using hexokinase 1 (HK1), and glucose-6-phosphate dehydrogenase (G6PD) assay kits (MSKBIO, China) and phosphofructokinase 1 (PFK1), phosphoglycerate kinase 1 (PGK1), α-enolase (ENO1), pyruvate kinase M2 (PKM2), and lactate dehydrogenase-1 (LDH1) assay kits (Bioswamp, Wuhan, China). Each measurement was performed in triplicate.

### 4.9. Quantification of Intracellular and Extracellular Lactate and ATP Production

For measuring lactate and ATP production, the cells were seeded at a density of 2 × 10^5^ cells/well in six-well plates and were allowed to adhere. The cells were treated with different concentrations of OBHS, 2-DG, metformin, and the combination on the next day and incubated for 24 h. The control group was prepared with 0.4 μL DMSO dissolved in 1999.6 μL corresponding medium. The culture medium and cells were collected, respectively. Cells were lysed by repeated freezing and thawing, and then centrifuged at 3000× *g* for 5 min at 4 °C; the supernatant was collected. Intracellular and extracellular lactate level and ATP production were determined using lactate assay kits (MSKBIO, Wuhan, China) and ATP assay kits (ELISA LAB, Wuhan, China), respectively. The experiments were performed in triplicate.

### 4.10. Reactive Oxygen Species (ROS) Determination

The intracellular ROS levels were measured using a 2′,7′-dichlorofluorescein diacetate (DCFH-DA) cellular ROS detection assay kit (Elabscience, Wuhan, China) following the manufacturer’s protocols. Briefly, cells were seeded into 96-well plates and treated as described previously. After OBHS treatment, cells were washed with PBS and stained with 10 µM DCFH-DA in a buffer for 1 h at 37 °C. After washing once with the buffer, ROS generation was analyzed using a fluorescent plate reader (SpectraMax iD5, Molecular Devices, San Jose, CA, USA). The absorbance was measured using excitation/emission wavelengths of 500/525 nm.

### 4.11. TdT-Mediated dUTP Nick-End Labeling (TUNEL) Staining

The air-dried cell slides were incubated with 4% paraformaldehyde (Beyotime Biotechnology, Shanghai, China) at 37 °C for 20 min. After the fixation step and washing with PBS, the cell slides were incubated with permeabilization solution (0.2% Triton X-100, Beyotime Biotechnology, Shanghai, China) at 37 °C for 10 min. The immunofluorescence staining of the breast cancer cells was performed using the One-step TUNEL in situ Apoptosis detection kit (FITC, Pricella, Wuhan, China) following the manufacturer’s instructions manual. Terminal Deoxynucleotidyl Transferase (TdT) equilibration buffer (100 µL) was added dropwise to each sample and incubated at 37 °C for 30 min. Next, 50 µL of TUNEL fluorescence detection solution was added to each sample and incubated at 37 °C for 60 min in the dark. Then, the sample was washed 3 times with PBS. Nuclei were counterstained with DAPI for 5 min at room temperature and washed 4 times with PBS. Observed the cortex from the cerebral under a laser confocal microscope (Leica TCS-SP8 SR, Wetzlar, German). The percentage of TUNEL/DAPI-positive apoptotic cells within the cortex were estimated based on the average number of cells in a defined area.

### 4.12. Transfection

sh-*SLC2A1* (PGPU6 shRNA) and sh-*pCU19* were synthesized by Hunan Fenghui Biotechnology (Changsha, China) and transfected into cells using Hieff Trans Liposomal Transfection Reagent following the manufacturer’s protocols. The cells were seeded in a six-well plate and reached 90–95% confluence before transfection. The cells recovered in 10% FBS-DMEM for 24 h. The transfection efficiencies were assessed by RT-qPCR and Western blotting 24 h after transfection. 

### 4.13. MCF-7 Xenograft Mouse Model

Four-week-old male athymic mice (nu/nu genotype, BALB/c background, purchased from Cavens (Changzhou, China)) with tumor formation were used as the model and were housed under aseptic conditions. Approximately 1 × 10^7^ MCF-7 cells were harvested, suspended in 200 μL of PBS and subcutaneously injected into the mammary pad of mice under anaesthetization by isoflurane. The mice were subsequently randomized into saline (*n* = 6, as vehicle control) and OBHS-treated groups (*n* = 6). They were then intragastrically administered 100 mg/kg of OBHS or the same volume of saline once daily for 7 consecutive days. The size of the subcutaneous tumors and weight of the mice were recorded daily. Tumor sizes were measured using Vernier calipers. Tumor volume was calculated according to the following formula: volume (mm^3^) = 0.5 × (length, mm) × (width, mm)^2^, where the length is the greatest diameter and the width is the diameter at the point perpendicular to the length. At the end of the treatment, the mice were sacrificed, and the tumor tissues from both groups were harvested and immersed and fixed in 4% paraformaldehyde before removal for immunohistochemical staining.

### 4.14. Immunohistochemistry

For immunohistochemical analysis, 0.3 cm thick tumor tissues of mice were isolated and fixed in 10% formalin for 48 h, followed by dehydration, paraffin embedding, and sectioning. Tissue sections of xenograft tumors (4 μm thick) were immunostained with the primary antibodies anti-Ki67 (1:300, Bioss, Beijing, China) and anti-GLUT1 (1:300, Bioss, Beijing, China) overnight at 4 °C. The peroxidase-conjugated streptavidin complex method was performed, followed by the 3,3′-diaminobenzidine procedure according to the manufacturer’s protocols. Images were captured using the Leica Application Suite imaging system (DM1000, Leica-Microsystems, Wetzlar, Germany).

### 4.15. Bioinformatic Studies

The Dependency Map (DepMap) portal is an open access to key cancer dependencies analytical and visualization tools whose goal is to empower the research community to make discoveries related to cancer vulnerabilities using the Broad Institute Cancer Cell Line Encyclopedia (CCLE) database (https://depmap.org/portal/interactive/, accessed on 4 April 2023). We selected the target cell lines in cell line selector, and these cell lines are indicated with an asterisk in subsequent analysis diagrams. 

Gene Expression Profiling Interactive Analysis (GEPIA) is an online tool for gene expression profiling interactive analysis (http://gepia.cancer-pku.cn/index.html, accessed on 4 April 2023). GEPIA can match the Genotype-Tissue Expression (GTEx) database and The Cancer Genome Atlas (TCGA) database to compare the gene expression in tumor tissue and normal tissue presented in boxplot. Gene Set Cancer Analysis (GSCA) is an integrated platform for genomic, pharmacogenomic, and immunogenomic gene set cancer analysis (http://bioinfo.life.hust.edu.cn/GSCA/#/, accessed on 4 April 2023). This database mainly integrates the drug resistance data of mRNA expression, mutation, immune infiltrates, methylation and Genomics of Drug Sensitivity in Cancer (GDSC) from the TCGA database. The expression analysis was performed using GEPIA and GSCA. 

Kaplan–Meier survival analysis was conducted using Kaplan–Meier plotter (KM plotter). KM plotter is an online survival analysis tool used to rapidly assess the effect of certain genes on cancer prognosis using microarray data. Overall survival (OS) and distant metastasis-free survival (DMFS) values for SLC2A1 were generated by KM Plotter for Breast Cancer (https://kmplot.com/analysis/index.php?p=service&cancer=breast, accessed on 4 April 2023).

### 4.16. Statistical Analysis

Each experiment was performed at least three times independently. Data are presented as the mean ± standard error (S.E.M.) for each experiment. Comparisons between two groups were performed using one-way analysis of variance (ANOVA), followed by the Bonferroni test, using OriginPro 8.0 software (Originlab, Northampton, MA, USA) and GraphPad Prism 9.0.0. A *p* value < 0.05 indicates a statistically significant difference.

## Data Availability

All data generated or analyzed during this study are included in this article.

## Abbreviations

WE: Warburg effect; OBHS: Oxabicycloheptene sulfonate; GLUTs: glucose transporters; GLUT1: glucose transporter 1; Akt: protein kinase B; mTOR: mammalian target of the rapamycin; SERM: selective estrogen receptor modulator; ER: estrogen receptor; 2-DG: 2-Deoxy-D-Glucose; DMSO: dimethylsulfoxide; FBS: fetal bovine serum; CCK-8: Cell Counting Kit-8; OS: overall survival; DMFS: distant metastasis-free survival; PVDF: polyvinylidene fluoride; Bcl-2: B-cell lymphoma-2; Bax: BCL2-Associated X; ECL: efficient chemiluminescence; GAPDH: glyceraldehyde-3-phosphate dehydrogenase; PBS: phosphate-buffered saline; OD: optical density; HK1: hexokinase 1; G6PD: glucose-6-phosphate dehydrogenase; PFK1: phosphofructokinase 1; PGK1: phosphoglycerate kinase 1; ENO1: α-enolase; PKM2: pyruvate kinase M2; LDH1: lactate dehydrogenase-1; DCFH-DA: 2′,7′-dichlorofluorescein diacetate; TUNEL: TdT-mediated dUTP Nick-End Labeling; TdT: Terminal Deoxynucleotidyl Transferase; GSCA: Gene Set Cancer Analysis; BRCA: breast cancer; TCGA: The Cancer Genome Atlas; GEPIA: Gene Expression Profiling Interactive Analysis; DEGs: Differentially expressed genes; SEM: Standard Error of Mean; PCA: Principal component analysis; PPP: pentose phosphate pathway; ROS: reactive oxygen species; PM: plasma membrane; MCT: monocarboxylate transporter.
